# Supplementary figures and images for: Comparative Proteomic Analysis Reveals Immune Competence in Hemolymph of Bombyx mori Pupa Parasitized by Silkworm Maggot Exorista sorbillans
Source: Insects. 2019 Nov 18;10(11):413. doi: 10.3390/insects10110413 (PMC6920964; doi:10.3390/insects10110413)

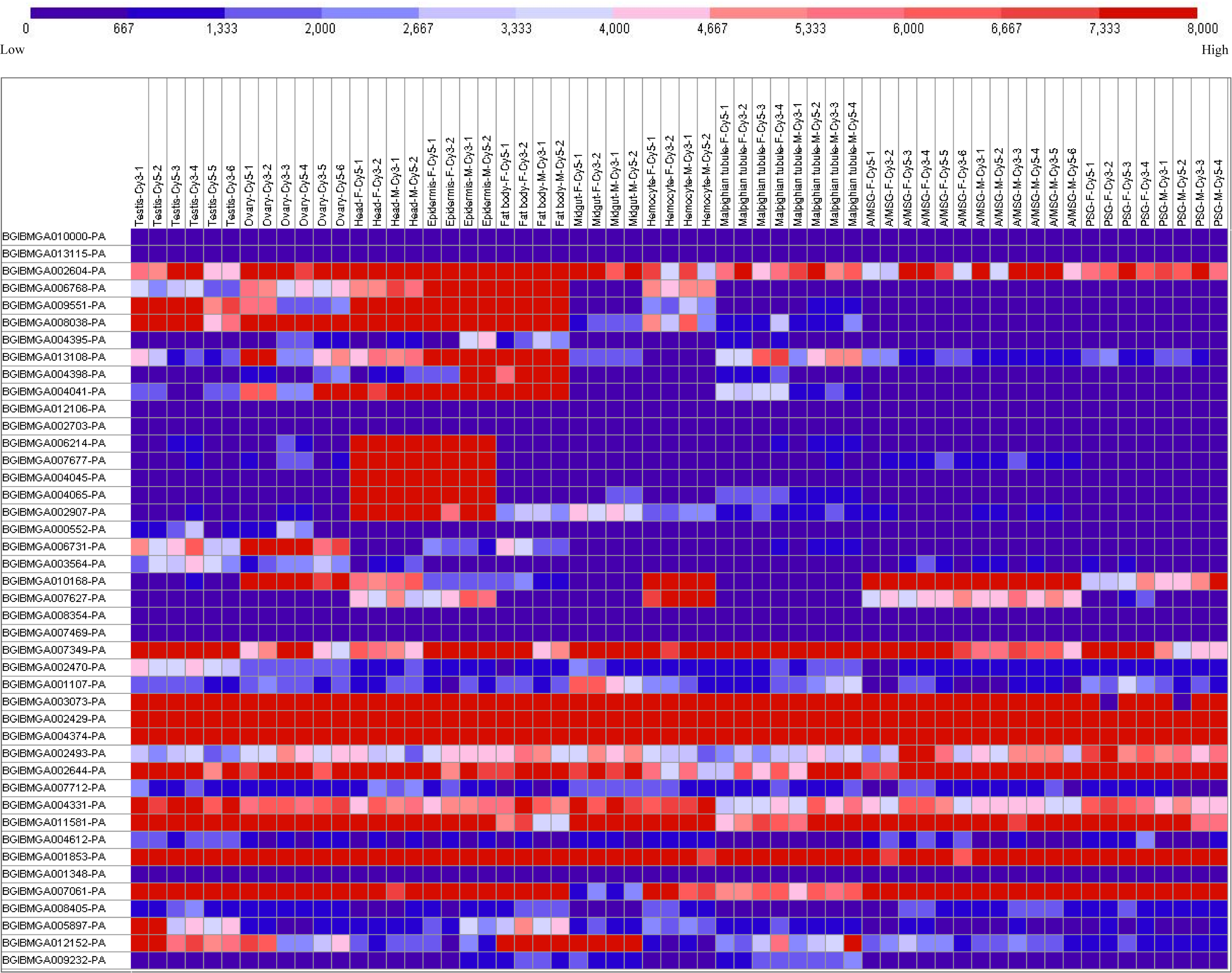

Supplement: Supplementary file 1 [file insects-10-00413-s001.zip › Supplementary/Figure S1.pdf]
